# *Sesame Street* Provides Lessons about Natural Brain Development in Children

**DOI:** 10.1371/journal.pbio.1001463

**Published:** 2013-01-03

**Authors:** Janelle Weaver

**Affiliations:** Freelance Science Writer, Carbondale, Colorado, United States of America

**Figure:**
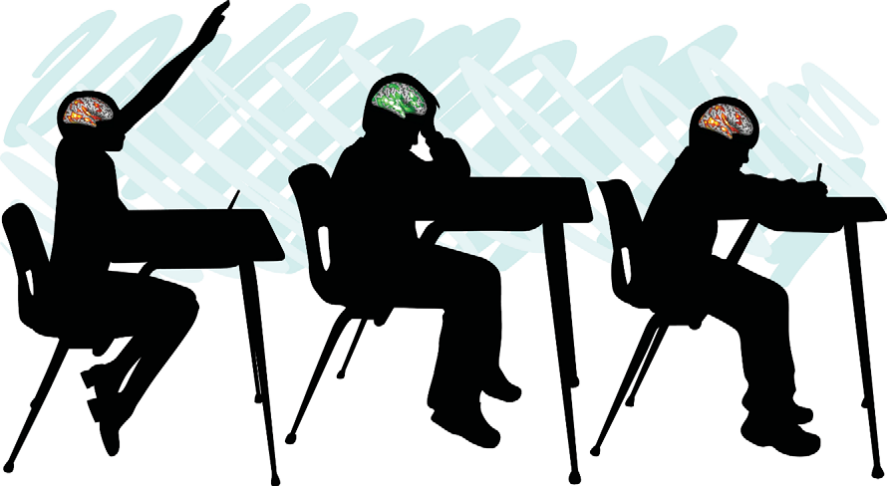
Children's school-based mathematics and verbal knowledge correlates with the maturity of their neural responses during the natural viewing of educational television programs such as *Sesame Street*.

Children are not the only ones who can learn a lesson or two from the Count. It turns out that neuroscientists are gaining key insights into brain development by turning to *Sesame Street*. While the vast majority of researchers who use neuroimaging techniques to study brain activity in humans use simple tasks and stripped down, short-lasting stimuli, such as pictures of isolated objects or individual tones, Jessica Cantlon and Rosi Li at the University of Rochester figured they could learn more about natural brain development by studying the neural activity of children as they watch real-world educational videos.

The results of this innovative study are published this week in *PLOS Biology*. Using functional magnetic resonance imaging (fMRI), Cantlon and Li found that the degree to which children showed adult-like brain responses while watching clips from *Sesame Street* predicted their performance on math and verbal IQ tests. The naturalistic neuroimaging method paves the way for future studies of brain development in the context of complex, real-world situations.

In the new study, 27 children between the ages of four and 11 and 20 adults participated in a series of fMRI experiments. These subjects first watched a 20-minute *Sesame Street* video consisting of clips covering a range of topics, including letters and numbers. Afterward, the children took standardized tests for mathematics and verbal IQ. The researchers then produced “neural maturity” maps by comparing the neural responses of children to those of adults.

The duo found that neural maturity in the intraparietal sulcus (IPS), a brain region involved in number processing, predicted children's performance on the math test. In other words, children with higher scores on the exam showed a more adult-like pattern of neural responses in the IPS while watching the educational video than did children with lower scores. On the other hand, neural maturity in a brain region known as Broca's area, which is involved in speech production, correlated with children's performance on the verbal IQ test.

The researchers went on to examine for the first time the relationship between children's brain activity during a traditional fMRI scan and math test performance. In the traditional fMRI experiment, the participants viewed pairs of faces, numbers, words, or shapes on a screen and reported whether or not they matched. In contrast to the results from the naturalistic experiment, the neural responses in the IPS did not predict children's math test performance in the traditional paradigm.

The findings indicate that neural responses to real-world stimuli are better predictors of math development than neural responses to simpler stimuli used in traditional fMRI experiments. One possible reason is that observing math-related lessons in educational videos is more relevant to school-based math experiences compared with matching pairs of numbers. In addition, natural viewing paradigms can reveal fluctuations in the timecourse of neural responses that aren't captured by traditional paradigms, which usually rely on short-lasting stimuli.

Taken together, the results suggest that the degree to which children's brains show adult-like patterns in their neural timecourses during natural viewing paradigms predicts their real-world academic performance. Moreover, the new fMRI method may be more effective than the traditional approach at linking patterns of brain development to children's experiences and performance in the classroom.

**Cantlon JF, Rosa Li R (2013) Neural Activity during Natural Viewing of** ***Sesame Street*** **Statistically Predicts Test Scores in Early Childhood. doi:10.1371/journal.pbio.1001462**

